# Experiences of caring for women with cervical cancer: A qualitative study among male partners in Dar es Salaam, Tanzania

**DOI:** 10.1111/hex.14038

**Published:** 2024-04-01

**Authors:** Rashid A. Gosse, Emanueli Amosi Msengi, Emmanuel Z. Chona, Joel S. Ambikile

**Affiliations:** ^1^ School of Nursing Muhimbili University of Health and Allied Sciences Dar es Salaam Tanzania; ^2^ Department of Clinical Nursing Muhimbili University of Health and Allied Sciences Dar es Salaam Tanzania

**Keywords:** caregiver burden, cervical cancer, lived experience, male partner, Tanzania, women

## Abstract

**Background:**

More than three‐fourths of cervical cancer cases occur in low‐ and middle‐income countries, with sub‐Saharan Africa (SSA) accounting for approximately 25% of global mortality. The significant rise in the prevalence of cervical cancer in SSA amplifies the burden on caregivers, contributing to elevated rates of mental illness, particularly among spouses who provide care. Men who assume the role of caregivers for their partners with cervical cancer encounter unique challenges and substantial adjustments across multiple facets of life, impacting both their own quality of life and that of their partners. Despite this, there is a notable lack of extensive research on the experiences of male partners in caregiving roles, particularly within SSA countries like Tanzania. Therefore, this study aimed to explore the experiences of male partners providing care for women with cervical cancer in Dar es Salaam, Tanzania.

**Methods:**

An exploratory qualitative study was undertaken to explore the experiences of 13 male partners, selected purposively and guided by the principle of saturation. Data gathering employed in‐depth interviews utilizing a semistructured interview guide, with subsequent analysis conducted via a thematic analysis approach.

**Results:**

Five themes and 13 subthemes were generated, encompassing psychosocial distress, attitudes towards cervical cancer, unity in the provision of care, economic burden, and altered sexual relationships. Participants reported experiencing emotional distress, shifts in social responsibilities, financial challenges, and unfulfilled sexual needs. Moreover, they expressed the need for social, psychological, financial, and sexual and reproductive support.

**Conclusion:**

This study underscores the numerous challenges encountered by male partners caring for women with cervical cancer, encompassing emotional distress, financial strain, and shifts in social and sexual dynamics. The identified themes and subthemes highlight the intricate interplay of these difficulties and stress the necessity for holistic support systems addressing the social, psychological, financial, and sexual aspects of male partners' experiences. The findings emphasize the importance of designing and implementing comprehensive support programmes tailored to the diverse needs of male partners, ultimately enhancing their quality of life and overall well‐being.

**Patient or Public Contribution:**

Before the study, the nursing manager assisted in selecting three male partners randomly. These partners were involved in the design of the participants' information sheet, the evaluation of the interview schedule and rooms, and the dissemination of information about the study's purpose to the target population. Their valuable input contributed to improving the participant information sheet, refining data collection procedures and addressing ethical considerations. However, these individuals were not considered study participants. Throughout the study, in‐charge nurses in the hospital were informed about the study's goals and helped organize appointments with participants and manage the interview schedule.

## 1 INTRODUCTION

Cervical cancer remains a global public health concern, affecting both women and men in various life aspects. The global burden of cervical cancer is unevenly distributed, with women living in low‐ and middle‐income countries (LMICs) and their families disproportionally affected.^1^ It was estimated in 2020 that LMICs contributed to nearly 90% of the global incidence and mortality of cervical cancer,^1^, ^2^ with a reported substantial increased burden in sub‐Saharan Africa (SSA), accounting for about 25% of global deaths.^3^ Tanzania is one of the countries in the SSA contributing the highest to the global burden of cervical cancer, whereby it is ranked fourth worldwide.^4^ The highest burden of cervical cancer in SSA is attributed to limited wide‐population cervical cancer screening and human papilloma virus (HPV) vaccination programmes, poor uptake of the existing cervical cancer screening and HPV vaccines, limited accessibility and affordability of the treatment, and a high prevalence of comorbidities such as human immunodeficiency virus (HIV) infection.^5^, ^6^, ^7^ In the SSA region, the majority of women with cervical cancer are diagnosed at late stages, hindering the effectiveness of treatment approaches and leading to a poor prognosis.^5^, ^6^, ^8^

Women diagnosed with cervical cancer experience significant life changes including physical suffering due to the disease itself and treatment side effects, emotional distress, and disruptions to various aspects of their socioeconomic lives.^9^, ^10^ Hence, it is crucial that they have responsible caregivers who can provide support and assist in coping with the life changes they encounter.^9^, ^11^, ^12^ In many African societies including Tanzania, men play a critical role in meeting socioeconomic needs and making health‐seeking decisions for their families. Traditionally, they are referred to as the primary family caregivers for their ill spouses.^13^, ^14^ Men involved in caring for their partners with cervical cancer encounter changes in psychological, socioeconomic, spiritual, and sexual aspects of their lives.^10^, ^15^, ^16^ Their experiences are mainly influenced by individual and social knowledge, attitudes, and perceptions of cervical cancer causes, symptoms and treatment.^17^, ^18^, ^19^ For instance, previous literature reports that the majority of men are not aware of the risk factors, signs and availability of cervical cancer treatment, and they perceive that their daughters and partners are at no risk for cervical cancer.^17^, ^19^ In some settings, men had linked cervical cancer to physical damage from frequent sexual practice and contraceptive use.^19^, ^20^ Socioeconomic burdens imposed by cancer treatment on caregivers trigger negative experiences such as feelings of being overwhelmed, fear of meeting the demands of the patient, and providing for the rest of the family.^21^, ^22^ The norms of masculinity also influence male partner experiences, and the majority of men had reported an increase in home‐based responsibilities and unmet sexual needs as the difficult aspects of caring for a partner with cancer.^23^

The new challenges and responsibilities imposed by a partner's illness may precipitate spousal caregiver burden, due to emotional stress, feelings of loneliness, changes in roles, altered sexual relationships, and increased financial demands.^10^, ^15^ Eventually decreasing the quality of life (QoL) of a caregiving partner.^15^, ^24^ It was also reported that as a disease advances, the caregiver of a patient with cancer may experience more psychological distress than that experienced by the patient.^25^ In SSA, caregivers' burden is another untold burden from cancer, which results in significantly high rates of mental health problems, commonly anxiety and depression, and an increased risk for chronic physical health problems.^26^, ^27^ An overwhelmed caregiver cannot provide support to the ill patient, and in some circumstances, the caregiver's distress triggers the patient's emotional distress.^10^, ^25^, ^26^ Hence, caregivers' burden may hinder the course of treatment and consequently worsen the patient's prognosis.^24^, ^26^

Exploring the experiences of male partners caring for patients with cervical cancer is crucial in providing valuable insights to support men in their caregiving roles and enhance support for partners during treatment and post discharge.^10^, ^27^, ^28^ However, in SSA, particularly Tanzania, information regarding the experiences, valuable care and supportive needs of men caring for their partners with cervical cancer is scarce. Therefore, to address the knowledge gap, the study focused on answering the question, ‘What are the experiences of men caring for their partners diagnosed with cervical cancer in Dar es Salaam, Tanzania?’ To inform ways to provide support to male caregivers and improve their QoL and overall well‐being.

## 2 MATERIALS AND METHODS

### 2.1 Study design and setting

Male partners' experiences of caring for women with cervical cancer were explored through an explorative‐qualitative study design. This approach facilitated an in‐depth exploration of the participants' experiences and obtaining detailed information as proposed by Doyle et al.^29^

The study was conducted at the Ocean Road Cancer Institute (ORCI), a specialized national referral hospital for cancer treatment located in Ilala City Municipal in Dar es Salaam, Tanzania. The hospital serves as a major centre for cancer detection, treatment, palliative care, and other supportive care in the country, with multidisciplinary and specialized health professionals providing care. It offers both inpatient and outpatient cancer services to patients from all regions of the country, admitting approximately 5500 new cancer patients annually. Among these newly admitted patients, 39% are diagnosed with cervical cancer. Treatments offered at the hospital include chemotherapy and radiotherapy. The institute receives cervical cancer patients across various age groups, with the majority falling within the range of 40–60 years of age. In terms of the stage of the disease, the majority of patients are diagnosed at stages II and III. The government of Tanzania, through the Ministry of Health, provides funding for radiotherapy treatments at the hospital. However, chemotherapy treatments are funded by patients themselves, either through their health insurance cards or out‐of‐pocket expenditures, of which the majority do not have health insurance cards.

### 2.2 Study population and eligibility criteria

The study population consisted of male partners of women diagnosed with cervical cancer and were receiving treatment at ORCI. The male partner in this study was referred to as a husband whose wife had cervical cancer and was involved in escorting his wife to the clinic or visiting her in the ward. The criterion for inclusion was being a male partner who had his wife diagnosed with cervical cancer for a timeframe of not less than a year. As the majority of caregivers of patients with chronic illnesses experience life changes and challenges as the disease progresses and express their experiences after feeling overwhelmed,^26^, ^30^ the time period from diagnosis was applied as one of the inclusion criteria to enhance obtaining rich information about participants' experiences. Male partners whose spouses needed support due to the severity of their conditions such as being taken for certain emergency services and those with known communication disorders that would interfere with participation, such as speech impairment, were excluded.

### 2.3 Sampling procedure and sample size

Study participants were recruited using a purposeful sampling technique. Male partners were approached for possible participation either at the wards during visiting hours or at the clinic while escorting their partners. Those who met the inclusion criteria and were willing to voluntarily share their experiences were selected for the interview. The interview took place at the participant's convenience and preferences; some consented to participate on the same day and others on the following days. Sample size determination was guided by the saturation principle as proposed by Guest et al.^31^ This measure ensured that the collected data supporting the study were sufficient while minimizing redundancy.^32^ We therefore continued to recruit participants for the interview until no new information could be obtained from them, which occurred with the 13th participant.

### 2.4 Data collection tool

We collected data using a semistructured interview guide that was developed based on the reviewed literature related to the study topic.^10^, ^15^, ^17^, ^28^ The guide was developed in English and then translated into Kiswahili, a national and widely spoken language, to facilitate understanding and communication between participants and researchers. Further, we developed a separate sheet to record participants' sociodemographic and background information. The interview guide was pretested 1 month before the actual study in the study setting, involving three male partners who met the inclusion criteria. This pretest aimed to ensure the understandability and appropriateness of the questions asked. Following the pretest results, any questions deemed unclear were modified, and inappropriate questions were omitted based on the feedback received. The information obtained from the pretested interviews was excluded from the actual study (to view the interview guide, see Appendix S1).

### 2.5 Data collection procedure

Data collection was carried out between December 2022 and March 2023 with the assistance of three male fourth‐year BSc. Nursing students. These research assistants were trained for 2 days to familiarize them with data collection tools, procedures, and interview‐sensitive topics. Interviews were carried out inside the prepared rooms available at the hospital premises, which provided privacy and enhanced participants' comfort and evenness during the interviews. During interviews, each question was followed by several probes to elicit further details and elucidate participants' responses. Each interview was audio‐recorded using a digital audio recorder, while other participants' information not captured by a recorder, such as nonverbal cues, was recorded in a notebook. Interviews lasted between 45 and 60 min.

### 2.6 Data analysis

Data analysis was done concurrently with data collection. Immediately following the first interview, data analysis was initiated by reviewing audio‐recorded data, doing verbatim transcription of the audio‐recorded information and translating the transcripts from Kiswahili into English.^33^ Translated transcripts were typed into Microsoft Word, and thematic analysis was done as proposed by Braun and Clarke.^34^ Specifically, each author read the transcripts iteratively with an open mind to get a general impression and familiarity with the data. The systematic meaning searching from transcripts and field notes was done to gain acquaintance with the experiences of male partners in caring for a partner with cervical cancer. The meaning units were identified from transcripts and were thereafter condensed to form codes. Different colours were used to highlight the patterns in the text conforming to the preconceived categories derived from the study objectives. Similar codes were ascertained and grouped together into subthemes reflecting the apparent meaning of the text. Then, similar subthemes indicating the underlying meaning of the text were organized into themes. Identified themes were reviewed by making comparisons across the transcripts to determine variation and correspondence with the study's perspectives. In every step of the analysis, reference was made to the audio data when a reflection and deeper understanding of the meaning units, codes, subthemes, and themes were required. To ensure the rigour and reflexivity of the findings, ongoing discussions among the authors were maintained throughout the data collection and analysis process, focusing on the meaning of the translated transcripts and the analysis outputs, such as subthemes and themes. Necessary modifications were made based on mutual agreements among the authors. Additionally, meetings were conducted with the interviewed participants to reach a consensus on the identified subthemes and themes. Participants' suggestions regarding the relevance and comprehensiveness of the findings were obtained and incorporated.

## 3 RESULTS

### 3.1 Sociodemographic characteristics

A total of 13 participants were included in this study, whose mean (SD) age was 55.5 (9.8) years, with six (46.1%) being in the age group 51–60. Most (38.4%) participants had partners who had lived with cervical cancer for 2 years. Eight (61.5%) participants had a primary education level, and the majority (76.9%) were Christians. Regarding the employment status, nine (69.2%) participants were self‐employed (Table 1).

**Table 1:** Participants' sociodemographic characteristics.

Variables	Frequency (*n*)	Percentage (%)
Age (in years)		
41–50	4	30.8
51–60	6	46.1
61–70	2	15.4
71–80	1	7.7
Level of education		
No formal education	2	15.4
Primary education	8	61.5
Secondary education	3	23.1
Religion		
Christian	10	76.9
Muslim	3	23.1
Employment status		
Formally employed	2	15.4
Self‐employed	9	69.2
Unemployed	2	15.4
Duration a partner lived with cervical cancer		
One year	4	30.8
Two years	5	38.4
Three years and above	4	30.8

### 3.2 Organization of themes and subthemes

Five themes and 13 subthemes were identified following thematic analysis. The themes identified comprised of psychosocial distress, attitudes towards cervical cancer, unity in provision of care, economic burden and altered sexual relationships (Table 2).

**Table 2:** Summary of themes and subthemes.

Themes	Subthemes
Psychosocial distress	Response to news about diagnosis
	Worries about the partner's prognosis
	Social disruption
Attitudes towards cervical cancer	Personal attitude
	Societal attitude
	Role played by healthcare providers
Unity in provision of care	Social support
	Family support
	Being concerned by the partner's wellbeing
Economic burden	Increased financial demands
	Disruption of economic activities
Altered sexual relationships	Unmet sexual needs
	Reproductive concerns

### 3.3 Psychosocial distress

Almost all participants reported that their female partners suffering from cervical cancer had affected them psychologically and socially. They verbalized their psychosocial experiences from the early days their partners experienced the symptoms (before treatment initiation) until they were hospitalized. The psychosocial experiences were explained under three subthemes: response to news about diagnosis, worries about the partner's prognosis, and social disruption.

#### 3.3.1 Response to news about diagnosis

Participants reported that they received information with sadness and were shocked the first time it was revealed that their partners had cervical cancer. Most participants stated that they experienced a loss of physical strength and heart palpitations. Others felt like they were paralysed and out of mind at the time the physician disclosed the results. These were greatly influenced by the perceived threat of cervical cancer and its symptoms, such as excessive bleeding, that female partners suffered. For instance, some participants stated:

I received with great sadness to hear that my wife has been diagnosed with cervical cancer, I was shocked. I suddenly lost strength, I felt as if my body was paralyzed for several minutes. I saw it as a dream because I never thought that there would be a day when a member of my family would get cancer, it was a disease I was only hearing about in the media. (Participant No. 12)

The blood was coming out like tap water and I was thinking how my wife will recover. That's what I was shocked when the doctor told me my wife have cervical cancer because I thought my wife would not be alive as the blood was coming out profusely. (Participant No. 2)

Further, some described that after the disclosure of the news about their partners being diagnosed with cervical cancer, there was no comforting information provided by healthcare providers (HCPs).

The doctor told us to leave after telling us the sad news. What I remember is that he told us to make a plan to start treatment, but as for comforting us, no … we left the room confused. I wish he could make us calm before we left … we didn't know where to start. (Participant No. 11)

#### 3.3.2 Worries about the partner's prognosis

Many participants verbalized that they were worried and had fear about their partners' condition. The physical changes that their female partners presented, such as bleeding and weight loss, and the negative attitudes towards cervical cancer in the community, made them worry about their partners' prognosis and fate. They thought it could be the end of life for their partners. One participant expressed his worries in the following manner:

My wife being diagnosed with cervical cancer has really affected my life because I was afraid, worried and sad about my wife's condition. The biggest concern is losing my wife because I am not sure if she will recover of this disease and people used to say that if you get this disease, you will not recover. (Participant No. 3)

Some participants expressed grappling with serious mental issues like depression and losing hope as they witnessed their partners' suffering. Additionally, some participants feared for the survival of their partners, believing that they were nearing death.

I have become a sad and depressed person because my wife has been sick for a long time. (Participant No. 4)

#### 3.3.3 Social disruption

Participants verbalized changes in their social lives. They felt lonely as much time was spent seeking health services, leaving them with minimal time to spend with friends and engage in social activities. Likewise, they experienced shifts in social roles and responsibilities. Participants found themselves taking on house tasks that were previously handled by their partners. Some described their families as transitioning to a single‐parent dynamic, as reported by one participant:

…. my responsibilities have changed because after my wife started to get sick, I have become a father and a mother as well, I am involved in cooking, washing and other household duties … the time to meet with relatives and friends has decreased because I am now spending a lot of time nursing my wife and serving my family. (Participant No. 5)

Participants also reported lifestyle changes, leading to low involvement in recreational activities and time spent hanging out with friends and family. Many reported feeling nostalgic for their previous lifestyles and expressed sadness regarding these changes. One participant stated:

After my wife started to get sick, my life has changed, before that I used to find time to go to recreational places, attend various concerts, participate in various social events, but as you know, now I am taking care of my ill wife … I have no time to go there. I am no longer living such life, even though I find it difficult but I have no way it has been like that. (Participant No. 13)

### 3.4 Attitudes towards cervical cancer

Under this theme, the subthemes included personal attitude, societal attitude, and the role played by HCPs. Participants shared personal and societal perspectives on cervical cancer, detailing their thoughts and perceptions of the disease. Additionally, they discussed how their communities viewed and understood cervical cancer. Negative attitudes towards cervical cancer were predominantly attributed to a lack of education and accurate information about the disease, leading to the proliferation of myths and misconceptions surrounding cervical cancer within their societies. They further described the role played by HCPs in addressing personal and societal attitudes towards cervical cancer.

#### 3.4.1 Personal attitude

Participants expressed their beliefs and perceptions of cervical cancer and life with a partner with the disease. Most of them thought cervical cancer was a dangerous disease with no cure, regardless of the stage of diagnosis. Most verbalized death as the fate of a cervical cancer patient within a short period of time. Moreover, some believed their families were not at risk of cervical cancer. Regarding available treatments, particularly chemotherapy and radiation therapy, they believed that these treatments could lead to harmful effects such as bodily dehydration and infertility. Still, some believed the side effects of chemotherapy and radiotherapy were worse than cervical cancer itself, as expressed by some participants:

When I think of my wife at that time, I was seeing her as a person who was going to die any time soon because I knew she would not recover according to what I was thinking about cancer. I knew that a person suffering from cancer cannot finish even three months before dying, and considering how my wife was bleeding profusely. I didn't know that if a person is diagnosed early with cervical cancer, she can get treatment and recover, I knew that if you get it, that's the end of your life story … I was thinking that this disease might be for a certain group of people because I had never heard before of any woman suffering from cervical cancer in my society. (Participant No. 9)

When I heard first that my wife is going to take cancer drugs (chemotherapy) and x‐rays (radiotherapy), I was concerned a lot because I heard that cancer treatment is dangerous than cancer itself and no one survive after receiving cancer treatment. (Participant No. 3)

Moreover, some participants held the belief that cervical cancer was caused by prolonged use of contraceptives and engaging in promiscuous behaviour, as one participant stated:

At first, I believed that cervical cancer is caused by vaginal injury due to having many lovers and using contraceptive methods for a long time. (Participant No. 10)

#### 3.4.2 Society attitude

In this study, participants described how cervical cancer and caring for a patient with the disease were perceived in their societies. They reported a wide range of societal beliefs when discussing the disease, including the impossibility of surviving cancer of any type. Besides, participants reported that many people in the community were not aware of the existing cervical cancer screening services, the presence of HPV vaccines, and curative measures. People were also discouraged from seeking medical treatment as they believed it was a waste of resources.

I have been hearing many people from my society talking that a person with cervical cancer cannot recover and ends up losing their life … mmmh that is not impossible … no one from that society knew before if such services (cervical cancer screening and HPV vaccines) ever existed… . (Participant No. 9).

People in my village used to say that when someone has cancer is better to leave them at home or search for traditional help than wasting a lot of money in the hospital for incurable disease. (Participant No. 7)

In some societies, cervical cancer was reported to be associated with a curse inflicted by traditional supernatural powers, as stated by one of the participants:

Since this disease (cervical cancer) is not common in the community I come from, some say we should ask forgiveness from the spirits of our ancestors because there may be spiritual things we have gone against, that is why my wife is suffering from this disease that does not exist in the community. Others, when I tell them that there are many women suffering from this disease, they don't believe it, and they insisted that I think carefully, if I make a mistake, I should correct it because I will lose my wife. (Participant No. 12)

#### 3.4.3 Role played by healthcare providers

Participants explained that the information provided by HCPs corrected their attitude and led to a shift in their perspectives regarding cervical cancer and its treatment. Learning that cervical cancer could be curable and witnessing improvement in their partners' prognosis following the initiation of medical treatment led participants to believe that cervical cancer is indeed curable. One participant reports:

The health workers help me a lot because they give me information about my wife's progress. I did not believe that this disease is curable and I did not believe that my wife will be fine as it is now. But the health workers were giving me information and making me understand about this disease. Also, the health workers were giving me information about the treatment being given to my wife. They have certainly been a great help to me and changed my attitude about cervical cancer … (Participant No. 3)

Participants in this study also reported that health education about cervical cancer provided by HCPs in their societies had changed the perspectives of many regarding the disease. Learning about cervical cancer screening, HPV vaccination, and available treatments played a pivotal role in changing societal attitudes and perceptions. Some participants reported hearing people talk about the importance of early detection of cervical cancer and available treatments following health education provided in their communities.

Health workers are also doing a great job to educate the community about this disease (cervical cancer) … . In my community, health workers came twice to provide education, they have helped a lot to change the society's attitudes towards cervical cancer. There was a time when I was here to follow up the progress of my wife, when I got back home I heard that health workers provided the education about cervical cancer and I was hearing people talking about the availability of vaccines, and the possibility of recovery if someone is diagnosed early. In fact, nowadays people are encouraging women to go for checkups and their cooperation with me has increased. (Participant No. 10)

### 3.5 Unity in the provision of care

Participants expressed how their friends, neighbours, and family members had supported them throughout the period of caring for their partners. Additionally, most participants reported the feelings they had about their partners and how they supported them in recovering from the disease. The unity experienced by participants fell under three subthemes encompassing social support, family support, and concern about the partner's wellbeing.

#### 3.5.1 Social support

Participants reported receiving support from social groups, friends and neighbours. Members of society assisted in covering treatment costs, both through individual contributions and via support from social groups or networks. Some provided support by asking participants about their partners' progress and providing comforting words. Despite being away from home most of the time, participants were involved in community activities, as articulated below:

My friends and neighbors have not isolated me, they are close to me and my wife, and they encourage me a lot since I started this movement to take care of my wife. They have been holding my hand and for some time they are helping me pay for medical expenses. I am close to my community, even when I am not at home we share social activities. There is a group there called KIKOBA, we joined with my wife since she has not been sick, they have helped us a lot to pay for medical expenses and other small things like soap, water, petroleum jelly … . (Participant No. 8)

#### 3.5.2 Family support

Male partners received support from their children and relatives. Family members provided support by paying treatment costs and encouraging participants and their ill partners. Whenever participants left their homes to visit their hospitalized female partners, their relatives took care of their children. In most cases, families joined their efforts to ensure the hospitalized family member received the treatment as planned, as expressed by one participant:

My sons have been involved in my wife's treatment to a large extent because since she started to get sick until today they have made every effort to ensure that she recovers. We have been around different hospitals until we got here, we have done a lot really. I also appreciate the efforts made by my brothers and my wife's family to make sure we unite together as a family to ensure she get all the necessaries to recover … . When I am not at home, my wife's sister stays with the children and protects the family. (Participant No. 2).

#### 3.5.3 Being concerned about the partner's wellbeing

Participants were concerned about the wellbeing of their partners and were motivated to support them. They did everything possible to ensure their partners recovered from the disease. Most reported that once they were aware that their partners could recover from the disease, they worked hard to cover the treatment costs, provided comfort and encouraged their partners to adhere to treatment. Some reported their masculine nature, making them feel the responsibility to support their partners. Some went as far as seeking spiritual support to beat the disease, as stated by one participant:

I have tried my best to take care of my wife so that she can recover, even though at first I saw that I was losing my money and a person cannot recover, this is because people used to say that if you get cancer, you cannot recover. But my spirit was pushing me to continue helping my wife to get treatment. Since my wife started to get sick, I have not left her, I have been with her side by side every day. I used to comfort her with encouraging words when she was desperate. I can say … mmh … I have been very helpful in my wife's treatment until now. I started to give her religious help when we were at home, I was calling pastors to come and pray for my wife at home so that she could recover … I also try to give money that enables my wife to receive treatment, I even doing activities that no one think I would done all to get money for her medical expenses. (Participant No. 5)

### 3.6 Economic burden

Participants reported facing financial challenges even before their partners were diagnosed with cervical cancer. However, their financial situation deteriorated following their partners' illnesses. They reported increased financial demands due to high treatment costs, leading to significant expenditures to cover medical expenses. As a result, they sometimes struggled to provide for the rest of the family. Spending a lot of time trying to find a suitable treatment for their partners also increased their financial hardship. The subthemes under this theme included increased financial demands and disruption of economic activities.

#### 3.6.1 Increased financial demands

Participants reported large amounts of family income being spent on treatment costs. At times, they found themselves unable to cover the basic cost of living for the rest of the family, as well as other developmental activities, due to the financial strain caused by the treatment expenses. Most reported their financial status being bad even before their partners got sick, with the situation becoming worse after they started seeking treatment. The cost of medical investigations, drugs, and other daily consumables was too high for them to afford, at times delaying treatment initiation as mentioned by participants:

My wife's cancer treatment has affected my life to a great extent because whatever we earn we spend on treatment. So my economy has fluctuated a lot since my wife started to get sick. Right now it has been different from the beginning because before my wife started getting sick, the money I was getting was doing various developmental things, but now the money I am getting ends up in treatment. (Participant No. 2)

… you know there are different costs here like drugs, x‐rays, and living expenses that needs money and considering my economic status sometimes I fail to pay on time and my wife miss important treatments … . (Participant No. 7)

Some participants resorted to selling their assets, such as land, to acquire funds to cover the expenses associated with treatment, as reported here:

My wife's illness has affected my economy to a great extent. The medical expenses have been high and burdened me considering I did not have much income. I had farms, I have sold almost three hectares … until now I have sold livestock and I still don't know where it will end up. I don't know what I will sell in the coming days because I see that the assets I had are about to vanish. (Participant No. 13)

#### 3.6.2 Disruption of economic activities

Many participants reported that seeking health services and visiting their partners at the hospital had reduced their working hours. This led to a decrease in their earnings and, on some occasions, led to cessation of their businesses, as stated by one of the participants:

The economy has also been affected because I was struggling to earn a living by doing small businesses during that period (before a female partner being diagnosed with cervical cancer), but for now it is about to stop because as you know I am going around different places to seek for treatment for my wife. So now my economic situation became very difficult and other businesses have already stopped. (Participant No. 1)

### 3.7 Altered sexual relationships

Participants reported altered sexual relationships with their partners, which were grouped into two subthemes, including unmet sexual needs and reproductive concerns.

#### 3.7.1 Unmet sexual needs

Participants shared how their partners' illnesses had interrupted their sexual lives. When their partners started to experience symptoms such as vaginal bleeding and pain, sexual life became a problem. Others verbalized that they were no longer happy and satisfied with their marriage as they struggled to share their sexual emotions. As a result, some male partners sought out other women to fulfil their sexual needs.

My sex life has been greatly affected for sure because after my wife was diagnosed with cervical cancer, we never had sex because a doctor advised not to have sex until when she will be fine. I can't say I am not struggling with my emotions, there is a time when I wish I get a woman to enjoy with and share sexual emotions. Sometimes I even think of having another woman or even marrying a second wife… (Participant No. 3)

#### 3.7.2 Reproductive concerns

Participants reported being worried about the fertility of their partners. Many believed that their partners would not be able to conceive even if they recovered from cervical cancer. The disease process and the treatment modalities (mostly radiotherapy) took away their hope of having children in the future with their ill partners. Some verbalized that their family plans regarding the number of children and spacing had been disrupted by their partners' illnesses.

My wife's illness makes me worry a lot about my family. Now I think she will not be able to get pregnant again due to this disease she has. The radiation she was exposed to have increased my anxiety as to whether she will actually be able to conceive, actually I think she won't be able to. My family plans are disrupted. Until now we have two children, we were hoping to have more children but now I have absolutely no hope of having more children. (Participant No. 11)

Most participants stated that the HCPs did not inquire about their reproductive concerns even though they needed psychosexual help and information regarding their partners' reproductive health, as verbalized by one participant:

… aaaahhh for sure doctors did not ask about our sexual and reproductive life … I wish they told me about the consequences of cervical cancer and the treatments that my wife receive on her reproductive health. (Participant No. 6)

## 4 DISCUSSION

Participants' ages ranged between 41 and 78 years, with the majority being in the economically active period of their lives. They had various experiences including psychological distress, unity in the provision of care, economic burden, and altered sexual relationships. Moreover, participants reported personal and societal attitudes towards cervical cancer and their needs during and after hospitalization.

Caring for partners with cervical cancer resulted in psychosocial distress among participants in this study. The psychological burden experienced by male partners during the disclosure of cervical cancer diagnostic results could be explained by limited awareness, negative attitudes, and perceptions of cancer in society. In many societies, cancer is perceived as a debilitating and life‐threatening condition, regardless of the type or stage at which it is revealed.^35^, ^36^, ^37^ This study also revealed that most of the individual participants and their societies believed that cervical cancer was incurable and meant an immense death. It had been reported previously that the psychological burden on the cancer patient caregiver can worsen than that faced by the patients, especially when the disease advances and eventually reduces QoL.^25^ Males who provided care to their spouses with cervical cancer in a study conducted in Norway expressed the need for psychological help from the HCPs. The study reported that participants encountered serious mental health problems, but health professionals' attention was solely to their partners.^15^ This evidences the importance of providing psychological support and empowering family caregivers, particularly male partners, in the course of caring for patients with cervical cancer.^16^, ^25^, ^28^ Experienced social disruptions, such as loneliness, changes in social roles and responsibilities, and lifestyle changes, could impose stress on caregivers.^15^, ^16^ Male partners of women with cervical cancer in a study conducted in Norway experienced serious social disruption as they reported being lonely and overwhelmed with social responsibilities.^15^ These studies show that providing social support to caregivers of patients with cervical cancer is among the priorities of care.^38^, ^39^

The study provided evidence that the antecedent attitude of male partners regarding cervical cancer may influence their physical, psychological, and emotional support for their partners. The notion that cervical cancer always meant a death sentence and transmission being linked to being unfaithful in marriage demotivated some male partners to provide full support to their wives. Participants perceived that their wives were at no risk for cervical cancer, which is consistent with the study conducted in Kenya, where the majority of men perceived that their female partners were at no risk for cervical cancer, and only 21.8% reported that they would support their women seeking health services or treatment in the case of a cancer diagnosis.^20^ The previous studies reported that a lack of spouse support can hinder cervical cancer screening uptake and access to treatment.^40^, ^41^ Men's education about cervical cancer in Uganda updated their knowledge and attitudes, increased their acceptance of cervical cancer screening and HPV vaccines, as well as their willingness to support their partners and daughters in obtaining cervical cancer services.^18^ The study also provided evidence that the majority of members of their societies believed that cervical cancer was incurable, and others linked it with the curse of the spirits. Participants reported that most members of society were unaware of the availability of HPV vaccines, cervical cancer screening and treatment. Negative attitudes towards cervical cancer in society can fuel myths and misconceptions, which in turn perpetuate stigma towards women suffering from cervical cancer and their families, and this may eventually hamper the effectiveness of cervical cancer prevention strategies.^42^, ^43^ Inline, a community study conducted in Ethiopia found that the majority of the participants in the study were not aware of cervical cancer, its risk factors or preventive measures.^44^ The study provides consistent evidence that had been reported previously regarding the significance of addressing society's attitudes and providing correct information about cervical cancer.^45^, ^46^ Participants also described the vital role played by HCPs in changing their individual attitudes and society's attitudes towards cervical cancer. Health information provided by HCPs helped to counteract negative attitudes and increased motivation to support female partners. Society members' attitudes were also halted following the provision of community health education about cervical cancer. This increased social support for male partners and moved society towards spreading correct information about the availability of HPV vaccines, cervical cancer screening services, and curative treatment upon early cervical cancer detection. This shows how health information provided by HCPs is always considered factual by the majority of society and the importance of providing health information to caregivers and promoting community health education in addressing the cervical cancer burden. Family caregivers in previous studies described how psychoeducational interventions improved their QoL and motivation towards caring for their relatives suffering from cancer.^47^ Community‐focused education programmes have also shown a positive impact on increasing cervical cancer awareness, uptake of HPV vaccines, cervical cancer screening, and support for women on cervical cancer treatment.^48^, ^49^

Participants described their experiences of family and social support and being concerned about their partners' wellbeing. Social support for family caregivers has a potential buffering effect on alleviating stress and promoting adaptive coping strategies.^50^, ^51^, ^52^ The formation of socially supportive groups that comprise family and nonfamily members can be effective in caring for patients with cancer.^53^, ^54^ Supporting the caregiver of a patient with cancer is considered an important aspect of preserving their psychological wellbeing.^12^ This care approach has been associated with improved QoL for the caregiver and the patient.^12^, ^28^ Awareness that cervical cancer is curable increased male partners' motivation to care for their spouses and advocate for treatment. They provide psychosocial support, financial support, and sometimes valuable information to the HCPs that may have an impact on the course of treatment.^17^, ^55^

Increased financial demands and disruptions of economic activities increased the economic burden among the study participants. High medical expenses prompted the sale of family assets and the rechanneling of household earnings to cover treatment costs. Time spent seeking treatment, visiting hospitalized partners, and doing home‐based activities also reduced male partners' engagement in economic activities, which consequently decreased earnings and led to the ceasing of their businesses. The situation aggravated financial constraints, ruined the family economy and caused further difficulties for participants to cater for the basic needs of their families, as had also been reported in previous studies.^56^ Family financial challenges had been reportedly contributing to a delay in the initiation of cervical cancer treatment and interfering with the course of treatment.^9^, ^11^, ^57^ As also reported previously, the government and stakeholders, such as charity organizations are key players in easing the accessibility and affordability of health insurance that covers treatment costs and granting financial aid to patients incapable of paying for their medical expenses.^56^

The study evidenced that male partners experienced altered sexual relationships as they reported unmet sexual needs and reproductive concerns. Spouses' physical sufferings such as vaginal bleeding and pain following intimate contact, led to feelings of discomfort, which contributed to a loss of sexual desire, and this triggered them to look for sexual gratification from other women. Participants were also concerned about the reproductive health of their partners; they feared their partners being infertile due to the physical damage posed by cervical cancer and treatment measures, particularly radiotherapy and chemotherapy. They were also concerned with postdischarge sexual practices and reproductive health. Challenges in sexual relationships were also revealed in a study by Solli et al. among male caregivers of cervical cancer patients, and they reported that they received little information from health professionals concerning postcancer sexuality.^15^ These sexual and reproductive concerns affect relationships, resulting in emotional stress, harsh treatment, and separation of families, which ultimately complicate cervical cancer treatment, as it has been reported in previous studies.^9^, ^58^ Vermeer et al. revealed that many cervical cancer patients and their partners experienced sexual dysfunction overly due to psychosexual distress, and participants expressed the need for psychosexual support from the HCPs, but the medical attention was mainly on physical functioning.^10^

The study limitations include the fact that the study was centred on a single public institution; therefore, the findings might not be generalizable to the whole population and might vary with the experiences of men whose partners are under treatment for cervical cancer in private hospitals and those not on treatment. The translation of the transcripts might have affected the interpretation of the interview content, as some words in the recording language (Kiswahili) had no exact meaning in English.

## 5 CONCLUSION AND RECOMMENDATIONS

Despite experiencing unity from family and social members, participants in our study reported a myriad of challenges, including emotional distress, social disruptions, financial hardships and unmet sexual and reproductive needs. They expressed an essential need for social, economic and psychosexual support in caring for their sick partners. The findings underscore the necessity of addressing the challenges faced by male partners in interventions targeting women with cervical cancer. This may be achieved by engaging with male partners and providing them with information and resources to meet their psychosocial, reproductive and sexual needs. Additionally, initiating programmes to economically empower and support male partners to cover the treatment costs of their sick partners should be prioritized, with considerations for the affordability of health insurance. Various stakeholders such as HCPs, governmental and nongovernmental organizations and development partners may play a crucial role in providing such support.

## AUTHOR CONTRIBUTIONS

**Rashid A. Gosse**: Conceptualization; investigation; writing—original draft; methodology; validation; visualization; writing—review and editing; software; formal analysis; project administration; data curation; resources. **Emanueli Amosi Msengi**: Conceptualization; investigation; writing—original draft; methodology; validation; visualization; writing—review and editing; formal analysis; data curation; project administration; resources; software. **Emmanuel Z. Chona**: Methodology; validation; visualization; writing—review and editing; writing—original draft; investigation; conceptualization; software; formal analysis; project administration; data curation; resources. **Joel S. Ambikile**: Supervision; data curation; formal analysis; project administration; writing—review and editing; conceptualization; methodology; investigation; resources.

## CONFLICT OF INTEREST STATEMENT

The authors declare no conflict of interest.

## ETHICS STATEMENT

The ethical clearance for this study was obtained from the Muhimbili University of Health and Allied Sciences (MUHAS) Institutional Review Board with reference number DA.282/298/01.C/1466. Permission to conduct the study was obtained from the ORCI administration with reference number 10/VOL.XXI111‐B. Written informed consent to participate in the study was obtained from each participant before the interview. Confidentiality of participants' information was strictly maintained. To further maintain confidentiality, no participants' identifiers were in the data collection guide, and the recorded audio data was stored well and will be destroyed following the completion of the study. Participation was fully voluntary, and the participants were informed of the freedom to withdraw from the study at any stage if they desired so, without any penalty.

## Supporting information

Supporting information.

## Data Availability

The data that support the findings of this study are available on request from the corresponding author. The data are not publicly available due to privacy or ethical restrictions. Data sets analysed during the current study are available from the corresponding author upon reasonable request.
