# Supplementary material for: Experiences of caring for women with cervical cancer: A qualitative study among male partners in Dar es Salaam, Tanzania
Source: Health Expect. 2024 Apr 1;27(2):e14038. doi: 10.1111/hex.14038 (PMC10985225; doi:10.1111/hex.14038)
Supplement: Supplementary file 1 — Supporting information. [file HEX-27-e14038-s001.docx]

## INTERVIEW GUIDE

**Experiences of Caring for Women with Cervical Cancer: A Qualitative Study among Male Partners in Dar es Salaam, Tanzania**

**Participant No………… Date……………………….**

**Part I: Socio-demographic characteristics of participants.**

1. Age (in years) ………...

2. Duration a partner lived with cervical cancer (in years)…………

3. Participant’s religion

a) Christian ( )

b) Muslim ( )

4. Participant’s highest level of education?

a) No formal education ( )

b) Primary education ( )

c) Secondary education ( )

d) College/higher education ( )

5. Participant’s employment status?

a) Formally employed ( )

b) Self-employed ( )

c) Unemployed ( )

d) Retired ( )

6. Duration a partner lived with cervical cancer………………………..

**Part II: Semi-structured interview guide**

**Note:** The open-ended questions below will provide a guide to the interviews but the probing questions will be asked where necessary to get in-depth information about a specific matter.

1. How was your reaction when you received the news of your partner being diagnosed with cervical cancer?
2. How have you been experiencing caring a partner living with cervical cancer?
3. How your life have been since your partner’s diagnosed with cervical cancer?
4. How you have been involving in your partner’s course of treatment?
5. How your society members perceive providing care to a partner diagnosed with cervical cancer?
6. How have you been experiencing healthcare services you receive while caring for your partner?
7. How your sexual relationship has been since your partner being diagnosed with cervical cancer?
8. How you have been supported while caring for you partner?
9. Please share anything more concerning your experience with caring for your partner that you would like to add?

**Thanks for your cooperation!**
